# SMYD3: a regulator of epigenetic and signaling pathways in cancer

**DOI:** 10.1186/s13148-021-01021-9

**Published:** 2021-02-26

**Authors:** Benjamin J. Bernard, Nupur Nigam, Kyunghee Burkitt, Vassiliki Saloura

**Affiliations:** 1 Thoracic and GI Malignancies Branch, Center for Cancer Research, National Cancer Institute, 41 Medlars Drive, Bethesda, MD 20852 USA; 2 Case Western Reserve University, Cleveland, OH USA

**Keywords:** SMYD3, SMYD3 cancer implications, Chromatin modifier, Histone methylation, Non-histone methylation

## Abstract

Chromatin modifiers and their implications in oncogenesis have been an exciting area of cancer research. These are enzymes that modify chromatin via post-translational modifications such as methylation, acetylation, sumoylation, phosphorylation, in addition to others. Depending on the modification, chromatin modifiers can either promote or repress transcription. SET and MYN-domain containing 3 (SMYD3) is a chromatin modifier that has been implicated in the development and progression of various cancer types. It was first reported to tri-methylate Histone 3 Lysine 4 (H3K4), a methylation mark known to promote transcription. However, since this discovery, other histone (H4K5 and H4K20, for example) and non-histone (VEGFR, HER2, MAP3K2, ER, and others) substrates of SMYD3 have been described, primarily in the context of cancer. This review aims to provide a background on basic characteristics of SMYD3, such as its protein structure and tissue expression profiles, discuss reported histone and non-histone substrates of SMYD3, and underscore prognostic and functional implications of SMYD3 in cancer. Finally, we briefly discuss ongoing efforts to develop inhibitors of SMYD3 for future therapeutic use. It is our hope that this review will help synthesize existing research on SMYD3 in an effort to propel future discovery.

## Background

DNA accessibility is a determinant of genetic expression in the human genome. If DNA is not accessible to key transcriptional factors, neither transcription nor translation, two central processes of genetic expression, can occur. DNA is packaged around histones (H2A, H2B, H3, H4) to form chromatin [1]. With the assistance of histone H1 and other proteins, chromatin can be further condensed into a more compact state. This compact, condensed chromatin state effectively silences genes by rendering DNA inaccessible to transcriptional machinery [1]. For genes to be activated and transcription to ultimately occur, the DNA must be rendered accessible to the transcriptional machinery, which occurs by loosening the interactions of histones with DNA [1]. This complex process requires assistance from many proteins and enzymes. Among these enzymes, chromatin modifiers function by modifying histones at specific amino acid residues, most commonly the histone tails [1]. These modifications include acetylation, methylation, phosphorylation, ubiquitylation, and sumoylation [1]. The sum of these modifications “write” the “histone code,” which ultimately dictates how a specific gene will be transcriptionally regulated [1]. This code is then “read” by proteins, the so called readers, which translate the code into a specific chromatin state: either an active state that permits access of transcriptional machinery or a repressed state that prevents access of transcriptional machinery [1].

One specific type of histone modification is histone methylation. Histone methylation marks are imprinted by histone methyltransferases and “erased” by demethylases [1]. The family of protein lysine methyltransferases (PKMT) is comprised of enzymes that mono-, di-, or tri-methylate lysine residues [1]. Methylation of lysine residues commonly occurs on Histone H3 (Histone H3 Lysine 4 (H3K4), Histone H3 Lysine 9 (H3K9), Histone H3 Lysine 27 (H3K27)) and Histone H4 (Histone H4 Lysine 5 (H4K5), Histone 4 Lysine 20 (H4K20)) [1]. These histone methylation marks can either activate or repress chromatin accessibility, depending on which histone amino acid residue is methylated [2]. This adds great functional diversity to the effects of histone methylation. For example, methylation of H3K9 is canonically repressive [3], while methylation of H3K4 is canonically activating [1, 4–6]. To add further functional diversity to these marks, lysine residues can be mono-, di-, or tri-methylated, and the degree of methylation confers specific effects [7]. Overall, methylation appears to be a more long-lasting epigenetic mark than other histone modifications such as acetylation and phosphorylation and can thus result in long-term epigenetic regulation at its action sites [8].

The SET and MYND-Domain (SMYD) family is a PKMT family comprised of proteins that alter chromatin accessibility and gene expression via methylation of, and interaction with, different histone and non-histone targets [6]. The SMYD family encompasses five discrete proteins, SMYD1-5, with reported functions in both normal and pathologic conditions. While the key feature of all SMYD family members was thought to be the methylation of H3K4, recent work has shown that each member may methylate other histone and non-histone substrates as well [7]. While the comprehensive functions of each of the SMYD family members remain unclear, these methyltransferases have been implicated in the pathogenesis of multiple cancer types, rendering them attractive anticancer drug targets [2, 9]. In this review, we focus on a specific member of the SMYD family, SMYD3, and we discuss its structure, tissue distribution, reported substrates, cancer-specific functions, and ongoing drug discovery efforts, with the goal to highlight its clinical implications and therapeutic possibilities.

## Materials and methods

We used the PubMed literature database to systematically interrogate and identify original research articles and literature reviews that investigate the SMYD protein family and protein structure, SMYD3 tissue expression profiles, prognostic implications and mechanisms of SMYD3 in cancer subtypes, and currently available SMYD3 inhibitors. We used the search terms “SMYD family,” “SMYD3,” “SMYD3 and Cancer” for literature review and to identify the above discussion areas. We interrogated SMYD3 among some of the most common cancer types worldwide (hepatocellular, colorectal, gastric, cervical, breast, ovarian, prostate, lung, pancreatic, esophageal, bladder, glioma). We focused on studies published in peer-reviewed journals. Additionally, we narrowed the scope to include articles that investigated clinicopathologic and mechanistic implications of SMYD3. However, some studies included were strictly clinicopathologic, some mechanistic, and some both. Applying the above search criteria, we found 89 eligible articles.

## Main text

### Structure of SMYD3

While there are five members of the SMYD methyltransferase family (SMYD1-5), we will focus on SMYD3 and point out structural differences with other SMYD members.

SMYD3 and the larger SMYD family has two conserved structural domains: the catalytic Su(var)3–9, Enhancer-of-zeste, and N-terminal Trithorax (SET) domain, which is split by a Myeloid-Nervy-DEAF1 (MYND) domain, and the C-Terminal Domain (CTD) [6]. The SET domain of SMYD3 is comprised of two sections: 1) the S-sequence, which may function as a cofactor binder as well as for protein–protein interactions, and 2) the core SET domain, which functions as the primary catalytic location [6, 10–13]. In close relation to the SET domain are two other domains: 1) the post-SET and 2) the SET-I, which assist in cofactor binding, substrate binding, and protein stabilization [11, 14–17]. Of note, SET domains often exist with a pre-SET domain as well; however, this is absent in all SMYD proteins [6]. The SMYD family has consistent sequence homology among all species [6].

The MYND domain is a zinc-finger motif that has particular affinity for proline-rich regions and helps conduct protein–protein interactions [6]. Even considering its location relative to the catalytic SET domain, the MYND domain does not play a role in substrate or cofactor binding [6]. In fact, Abu-Farha et al. demonstrated that deletion of the MYND domain does not alter the methyltransferase activity of SMYD2 in vitro, highlighting that the MYND domain is not required for methylation [18]. Further, the MYND domain is positively charged, which in most cases allows for binding to negatively charged DNA. However, SMYD3 is the only member of the family which has been shown to directly bind to DNA in vitro [6].

SMYD3 contains a CTD, whose function is not fully elucidated. However, protein crystallography of SMYD1-3 seems to highlight that the CTD domain may have an enzymatic regulatory function by both enhancing and inhibiting the proteins’ methyltransferase activity [6, 10]. For example, shortening the CTD increases SMYD1 and SMYD2 activity, while a similar shortening inhibited SMYD3-induced methylation of histone H4 [11, 14]. These findings highlight the heterogeneity of the CTD as well as the necessity for ongoing research to elucidate its function.

In terms of structural comparisons, SMYD1-3 has similar overall structures and locations of their SET domains. The SET domain of SMYD4 is closer to the CTD than the other SMYD proteins, and its N-terminal domain contains Tetratricopeptide Repeats (TPR), a region structurally similar to the CTD [6]. Lastly, SMYD5 has a larger SET domain as well as being the only member of the SMYD family to lack a CTD. For a more detailed, comparative description of the structure of SMYD family members, we refer the reader to an excellent review by Tracy et al. [6].

### Tissue expression and normal functions of SMYD3

Kim et al. generated mass spectrometry data to analyze tissue expression patterns of the SMYD family in normal adult and fetal human tissues [19]. In fetal tissues, SMYD3 is highly expressed in the heart, moderately expressed in the ovary and brain, lowly expressed in the gut and testis, and not expressed in the liver. In adult tissues, SMYD3 is highly expressed in platelets and testis, moderately expressed in CD8 + T-cells, and lowly expressed in the frontal cortex of the brain, spinal cord, retina, heart, ovary, bladder, and prostate [19]. In adult tissues, SMYD3 is not expressed in the liver, lung, colon, rectum, and kidney, adrenal gland, gallbladder, pancreas, esophagus, bladder, prostate, or monocytes [19].

While much of the literature as well as the scope of this paper is focused on the role of SMYD3 in cancer, SMYD3 has been shown to be critical in cardiac and skeletal muscle development [6]. In fact, zebrafish embryos where SMYD3 was knocked down experienced cardiac defects and pericardial edema, pointing to a potential function of SMYD3 in cardiac development [6, 20]. Also, reduction of Smyd3 in C2C12 mouse myoblasts reduced expression of *myostatin* and *c-Met* genes, resulted in hypertrophic myotubes, and prevented dexamethasone-induced skeletal muscle atrophy in a mouse model [6, 21]. Furthermore, Codato et al. showed that Smyd3 overexpression promoted muscle differentiation and myotube fusion in C2C12 murine myoblasts [22]. Additionally, RNA expression analysis of Smyd3-overexpressing murine myoblasts showed a significant upregulation of genes associated with myogenesis (*Mck, Mymk, Tnnc1, Myh3, Myl4, Atp2a1*) [22]. The authors found that Smyd3, likely via H3K4 methylation, plays a role in the transcriptional regulation of a transcription factor called *myogenin* that is critical for muscle development during embryogenesis and throughout the lifespan [22]. These results underscore the role of SMYD3 in cardiac and skeletal muscle physiology. However, further investigation into the functions of SMYD3 in normal states and in human cell systems is critical.

### Histone and non-histone substrates of SMYD3

Over the past 20 years, a significant amount of preclinical work has unveiled that SMYD3 methylates both histone and non-histone substrates. This section briefly highlights some of the reported substrates of SMYD3. In the next section (“Cancer Implications”) we will review the implications of these SMYD3 substrates in cancer development and progression.

The first study to report SMYD3 as a methyltransferase was conducted by Hamamoto et al., demonstrating that SMYD3 di- and tri-methylates H3K4 in vitro [23]*.* They used 293 T cells transfected with plasmids expressing Flag-tagged wild-type SMYD3 and enzymatically inactive SMYD3, and tagged proteins were purified by immunoprecipitation using a Flag-targeting antibody [23]. These immunoprecipitates were co-incubated with recombinant histone H3 and 3H-labeled S-adenosyl-L-methionine (SAM) in an in vitro histone methyltransferase assay and blotting of the reactants identified H3K4 di- and tri-methylation as enzyme end products of wild-type SMYD3 [23]. Foreman et al. showed that SMYD3 preferentially tri-methylates H4K20, a transcriptionally repressive mark [10]. Similarly, this group utilized an in vitro system of co-incubated immunoprecipitated SMYD3 with recombinant H4 and radio-labeled SAM in 293 T cells [10]. Furthermore, Van Aller et al. first demonstrated that SMYD3 primarily mono-methylates H4K5 rather than H3K4 and H4K20, using an in vitro methyltransferase where histone peptides, recombinant histones, or recombinant nucleosomes were co-incubated with SMYD3 (wild-type or SMYD3 mutants) and SAM [24]. The results were then analyzed using liquid chromatography or mass spectrometry analysis [24]. Interestingly, these studies show that SMYD3 methylates both activating (H3K4) as well as repressive marks (H4K5/H4K20). Further investigation is needed to elucidate the histone substrates of SMYD3, given that the above assays were predominantly conducted using recombinant substrates and nucleosomes which may not necessarily capture the three-dimensional conformation of chromatin in living cells. Additionally, it would be important to decipher whether SMYD3 has a preferential effect on H3K4, H4K20, or H4K5 based on the cell context or whether methylation of these substrates occurs concurrently at variable levels in living cells.

**Fig. 1:**
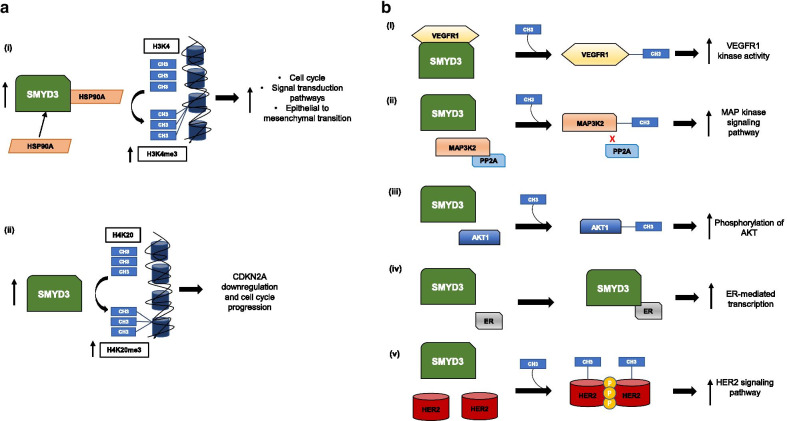
Selected mechanisms of action of SMYD3 as an oncogenic driver. **a** Histone-mediated mechanisms. (i) SMYD3 tri-methylates H3K4, with HSP90A enhancing its enzymatic activity. (ii) SMYD3 tri-methylates H4K20. In ovarian cancer cells, CDKN2A is repressed via SMYD3-mediated H4K20 tri-methylation. **b** Non-Histone-mediated mechanisms. (i) SMYD3 methylates vascular endothelial growth factor receptor 1 (VEGFR1) at lysine 831 and enhances its kinase activity. (ii) SMYD3 methylates mitogen-activated protein kinase kinase kinase 2 (MAP3K2) at lysine 260, preventing its dephosphorylation by protein phosphatase 2 (PP2A) and activating the MAP kinase pathway. (iii) SMYD3 methylates v-Akt murine thymoma viral oncogene homolog 1 (AKT1) at lysine 14 and increases its phosphorylation and activation. (iv) SMYD3 acts as a co-activator of the estrogen receptor (ER), increasing the transcription of ER-mediated downstream genes. (v) SMYD3 methylates human epidermal growth factor receptor 2 (HER2) at lysine 175, enhancing HER2 homodimerization and autophosphorylation

SMYD3 has been shown to methylate non-histone targets as well, specifically the Vascular Endothelial Growth Factor Receptor 1 (VEGFR1), MAP3 Kinase 2 (MAP3K2), AKT1, Estrogen Receptor (ER), and Human Epidermal Growth Factor Receptor 2 (HER2), in addition to others [25]. These specific interactions and the cancer types in which they were studied will be discussed in greater depth in the next section. VEGFR1, a receptor tyrosine kinase that plays a crucial role in angiogenesis, has been shown to be methylated by SMYD3 at lysine 831, which enhances its kinase function [26]. Additionally, MAP3K2 is a protein kinase that is a member of the Ras family of oncogenes, well-known to be activated in a large proportion of cancers. Mazur et al. have shown that SMYD3 directly methylates MAP3K2 at lysine 260, and this enhances activation of the Ras/Raf/MEK/ERK signaling pathway [27]. Moreover, AKT1, a serine-threonine kinase, is a key mediator of a pathway necessary for cell growth, survival, glucose metabolism, and neovascularization [28]. Yoshioka et al. demonstrated that SMYD3 methylates lysine 14 of AKT1, and this is a critical step required for AKT1 activation [28]. Furthermore, SMYD3 has been shown to interact with the estrogen receptor (ER) [29]. The ER-SMYD3 complex is recruited to the regulatory regions of ER target genes and has been shown to enhance transcription [29]. Lastly, HER2, a receptor tyrosine kinase, is overexpressed in a subset of cancers [30]. Yoshioka et al. demonstrated that SMYD3 tri-methylates HER2 at lysine 175, which enhances HER2 homodimerization and activation [30]. Table 1 lists these histone and non-histone substrates of SMYD3, and Fig. 1 graphically illustrates these important oncogenic mechanisms.

**Table 1:** SMYD3 Histone and Non-Histone Substrates

Substrate	Enzyme endproduct	References
*Histone substrates*		
H3K4	Di- and Tri-methylation of H3K4	[23]
H4K5	Mono-methylation (to the greatest degree), di-methylation, and tri-methylation of H4K5	[24]
H4K20	Tri-methylation of H4K20	[10]
*Non-histone substrates*		
VEGFR1 (vascular endothelial growth factor receptor 1)	Methylation of VEGFR1 at lysine 831	[26]
MAP3K2 (MAP 3 Kinase 2)	Mono-, di-, or tri-methylation of MAP3K2 at lysine 260	[27]
AKT1	Methylation of AKT1 at lysine 14	[28]
ER (estrogen receptor)	Transcriptional coactivator of ER	[29]
HER2 (human epidermal growth factor receptor 2)	Tri-methylation of HER2 at lysine 175	[30]

### Interacting proteins of SMYD3

Heat shock protein 90 (HSP90), a nuclear chaperone, has been shown to be critical for the nuclear localization and function of SMYD3 [12, 23, 31]. Brown et al. used in vitro and in vivo methods to investigate HSP90 binding to the CTD of SMYD3 and how this interaction facilitates SMYD3 nuclear localization [14]. After analyzing the 3-dimensional conformation of the CTD of SMYD3 and HSP90, the authors hypothesized that the CTD and HSP90 may bind [14]. In vitro, the authors generated SMYD3 mutants where SMYD3 was truncated at the CTD and performed HSP90 binding assays (varying amounts of HSP90 mixed with SMYD3 proteins in a reaction and centrifugated pellets of SMYD3 alone, HSP90 alone, and SMYD3-HSP90 complexes) in addition to histone methyltransferase assays using recombinant histone, HSP90, radio-labeled SAM, and SMYD3 [14]. The authors found that truncated SMYD3 led to decreased HSP90 binding which in turn led to decreased methylation of H3K4. To confirm the in vitro effects of SMYD3 truncated at the CTD in an in vivo model, overexpressed FLAG-tagged SMYD3 constructs were evaluated in NIH3T3 fibroblasts [14]. These constructs underwent nuclear/cytoplasmic fractionation, and when SMYD3 was truncated at the CTD, nuclear localization did not occur. This highlighted that HSP90 is critical for nuclear localization of SMYD3 [14]. Further, they performed a proliferation assay in murine embryonic fibroblasts comparing wild-type SMYD3 to the SMYD3 mutant and found that the SMYD3 mutants had significantly decreased proliferation when compared to wild-type SMYD3 [14]. In an in vitro methyltransferase assay, Hamamoto et al. found that methylation of histone H3 by wild-type SMYD3 increased in a dose-dependent manner when combined with recombinant HSP90 [23]. These findings suggest that HSP90 binding to SMYD3 not only assists in localization, but also is necessary for the methyltransferase activity of SMYD3.

Further, Hamamoto et al. demonstrated via immunoprecipitation that SMYD3 forms a complex with RNA polymerase II and RNA helicase, whereby RNA helicase serves as a bridge [23]. This is a critical step for transcriptional elongation [23]. Further research is necessary to characterize the full spectrum of interacting proteins of SMYD3, as well as the variability of its interactions based on the cell-specific context.

### Implications of SMYD3 in cancer

While the function of SMYD3 has been less explored in normal tissues [6, 20–22], much of the investigation of SMYD3 has focused on its role in carcinogenesis and tumorigenesis. Reported roles of SMYD3 in cancer include the following: epithelial-mesenchymal transition, cell cycle alteration, promotion of cell proliferation, increased telomerase activity, and cell immortalization [32–35]. In this section, we will review reported functions of SMYD3 in various cancer types. Table 2 summarizes the clinicopathologic and mechanistic findings pertaining to SMYD3 in the cancer types described in greater detail below.

**Table 2:** Cancer-specific implications of SMYD3

Cancer type	Clinicopathologic associations	Cancer cell phenotypes	Molecular mechanisms
Colorectal cancer (CRC)	SMYD3 expression correlates with: incidence of CRC [36], advanced T stage [38], and lower survival rates [38]	Cellular Proliferation and EMT [36]	JAK/STAT pathway and MYC/Ctnnb1 oncogenes [36]
	Lower SMYD3 promoter methylation levels correlate with lymph node metastasis and stage III/IV disease [37]		
	SMYD3 is identified as an independent prognostic factor in CRC [38]		
Hepatocellular carcinoma (HCC)	SMYD3 expression correlates with: HCC development [36], shorter overall and disease-free survival after chemotherapy [36, 39], and poor prognosis in stage I-II disease [2]	Cellular proliferation (via regulation of CcnA2, CcnE1, CcnD1, Pcna, Igfbp1, CK2, MMP2) [2, 36]	HSP90a enhances SMYD3-mediated H3K4 methylation [23]
		EMT (via regulation of Snai1, Snai2, Twist, Zeb1, SOX4, Fn1, Vimentin, Timp1, Mmp2, Mmp7, Mmp9, and Mmp14) [23, 36]	Transcriptional elongation (via recruitment of helicase and RNA polymerase II) [23]
	SMYD3 is an independent prognostic factor of 5-year HCC relapse [2]		SMYD3-HBX Interactions (activation of AP-1, PI3K/AKT and ERK signaling, C-MYC) [40, 41, 43]
Breast cancer (BC)	Decreased SMYD3 mRNA expression is associated with improved relapse-free survival [44]	EMT (via regulation of TGF-beta controlled EMT-specific transcription factors such as SMAD3) [47]	Upregulation of WNT10B [45, 46]
			Tri-methylation and activation of HER2 [30]
		Cell proliferation (via expression changes in cell cycle genes (Cyclin D1, CDK2, and CDK4) and apoptosis genes (such as p53/p21 and Bcl-2/BAX ) [51]	Methylation of VEGFR1 leading to its enhanced kinase activity [26]
			Transcriptional coactivation of ER, and enhancement of ER-mediated transcription [29]
Gastric carcinoma (GC)	SMYD3 expression correlates with: larger size of primary tumor, greater lymph node metastasis, and advanced TNM stage [52], and Poorer 5-year survival [52]	Cell proliferation (via increased G2/M progression mediated by ATM-CHK2/p53) [58]	Beta-catenin/TCF4/SMYD3-mediated ASCL2 activation [53]
			-SMYD3-STAT3 interaction [54]
Cervical cancer		Cell proliferation [59]	
Prostate cancer	SMYD3, and EZH2 independently predict prostate cancer patient outcome [60]		SMYD3-mediated H4K20 tri-methylation, and repression, of CCND2 [61]
			SMYD3-mediated binding and regulation of AR [63]
Pancreatic cancer/lung cancer	SMYD3 expression correlates with: tumor size, TNM stage, perineural invasion, lymph node metastasis in pancreatic adenocarcinoma, and shorter overall survival [65]		SMYD3-mediated mono-, di-, or tri-methylation of MAP3K2 [27]
	SMYD3 was identified as an independent prognostic indicator for overall survival [65]		SMYD3-dependent MAP3K2 tri-methylation blocks MAP3K2 from inactivation by PP2A [27]
Non-small cell lung cancer (NSCLC)	SMYD3 expression correlates with: never-smoked history, advanced pathologic stage, larger tumor size, presence of lymphovascular invasion, pleural invasion, distant metastasis [70], and poorer disease free-survival and overall survival [70]	Cell proliferation (Via induction of apoptosis via regulation of Bim, Bak, Bax, BCL-2, and Bcl-xl [70]	SMYD3-mediated cisplatin resistance [72]
		EMT (via regulation of MMP-2 and MMP-9) [70]	HSP90 serves as a co-factor in SMYD3-mediated transcription of Met [71]
Ovarian cancer	SMYD3 expression increases from normal ovaries → fallopian tubes → primary cancer lesions → metastatic lesions [72]	EMT (via regulation of E-cadherin and vimentin [72]	SMYD3 and UBE2R2 interaction promotes ubiquitination/degradation of p53 [72]
	SMYD3 expression correlates with increased risk of lymph node metastasis and poor overall survival [72]	Cellular adhesion (SMYD3-mediated binding to *ITGB6* and *ITGAM)* [75]	Increased BIRC3 expression via SMYD3-mediated H3K4 tri-methylation [73]
			Decreased CDKN2A expression via SMYD3-mediated H4K20 tri-methylation [73]
Esophageal cancer	SMYD3 expression correlates with increased risk of lymph node metastasis and poor overall survival [76]		SMYD3-mediated binding to, and transcription regulation of, *EZR *and *LOXL2* [76]
	SMYD3 is an independent prognostic factor for poor overall survival [76]		SMYD3-EZR-AS1 interaction enhances EZR transcription [77]
	Patients with the SMYD3 3/3 VNTR genotype had a 3-fold increase of esophageal squamous cell carcinoma when smoking, but no increase in non-smokers [78]		
Bladder cancer	SMYD3 expression correlates with: tumor stage, lymph node metastasis, and poorer progression-free survival [81, 84], shorter overall survival [81, 84]	Cell proliferation (Increased apoptosis via regulation of Cyclin D1, CDK4, Cyclin E1, CDK2, p21, and p27 [81]	Possible feedback loop of SMYD3/IGF-1R/AKT/E2F-1 [81]
		Autophagy activation (via BCLAF1 interaction) [84]	SMYD3 interacts with, and modulates expression of, BCLAF1 via H3K4 di-/tri-methylation [84]
Malignant Glioma	SMYD3 expression correlates with: poorer overall survival [87]	Cell proliferation (possibly via regulation of p53) [87]	
	SMYD3 expression inversely correlates with: p53 expression [87]		

#### **Colorectal cancer**

To investigate the role of SMYD3 in colorectal cancer (CRC) and hepatocellular carcinoma (HCC) formation, Sarris et al. generated a mouse model of Smyd-deficient, overexpressing, or wild-type mice (C57BL/6) and exposed them to diethylnitrosamine (DEN) or 1,2-dimethylhydrazine/dextran sodium sulfate (DMH/DSS), chemicals known to induce CRC and HCC and in wild-type mice of this strain [36]. Prior to exposure to DEN or DMH/DSS, there were no histologic or macroscopic differences among the groups, which indicated that Smyd3 was not a requirement for normal colon or liver development [36]. When the mice were exposed to DEN/DMH/DSS treatments that induce liver or colon cancer, there was a dramatic reduction in gross tumor size and number in the Smyd3-deficient group with only a few focal nodular hyperplastic areas detected histologically in the DEN-treated Smyd3-deficient mice. Furthermore, the DEN-treated wild-type and overexpressing mice had similar tumor sizes and numbers [36]. In this study, the authors concluded that Smyd3 expression in mice is required for chemically induced CRC and HCC formation. Additionally, tumorigenesis likely occurred through transcriptional activation of regulators of cell proliferation, epithelial-mesenchymal transition, JAK/Stat3 pathway, and Myc/Ctnnb1 oncogenes (further discussed in HCC section) [36].

In another study, Li et al. investigated the association between *SMYD3* promoter methylation and CRC in a Chinese cohort using quantitative methylation-specific PCR (qMSP) [37]. The study analyzed 117 CRC tumor tissues and paired normal tissues [37]. Results demonstrated that tumor tissues had significantly lower *SMYD3* promoter methylation levels than adjacent normal tissues [37]. Subgroup analysis showed that significantly lower *SMYD3* promoter methylation was observed in CRC patients with lymph node (LN) metastasis and stage III/IV disease [37]. Lastly, The Cancer Genome Atlas (TCGA) analysis verified an inverse correlation between *SMYD3* methylation and *SMYD3* expression. These results highlight that *SMYD3* promoter hypomethylation may be a mechanism of SMYD3 overexpression, resulting in carcinogenesis and tumor progression in CRC.

Liu et al. investigated the prognostic significance of SMYD3 expression in CRC [38] using immunohistochemistry (IHC). In total, 173 patients diagnosed with stage I-III CRC were included in the study [38]. High SMYD3 expression was significantly associated with advanced tumor stage and was identified as an independent prognostic factor of poor survival in patients with CRC [38].

These findings support that SMYD3 plays a critical role in CRC carcinogenesis, promoter hypomethylation may be a mechanism for SMYD3 upregulation, and that SMYD3 confers lower a survival rate and poor prognosis in this disease. Further mechanistic investigation is warranted to evaluate SMYD3 as a therapeutic target in CRC.

#### Hepatocellular carcinoma

Hamamoto et al. published a seminal study describing SMYD3 as a histone methyltransferase with oncogenic functions in HCC and CRC [23]. They showed that SMYD3 is overexpressed in both HCC and CRC tumors and that SMYD3 knockdown inhibits the growth of HCC and CRC cells [23]. They then demonstrated that SMYD3 carries out these functions by altering transcription in two critical ways: 1) increased chromatin accessibility leading to increased promoter accessibility by methylation of H3K4, 2) transcription elongation by RNA Pol II recruitment [23]. They further explored that RNA helicase serves as a bridging protein between SMYD3 and RNA Pol II [23]. Additionally, they demonstrated that HSP90A enhances the enzymatic activity of SMYD3 [23].

In another seminal study by Sarris et al. [36], analysis of SMYD3 mRNA levels in the TCGA database for HCC revealed significantly higher expression levels of SMYD3 in HCC compared to normal liver tissues, which was corroborated by the TCGA database [36]. Kaplan–Meier analysis demonstrated that patients with high tumor levels of SMYD3 mRNA had poorer overall survival [36]. Based on these findings, Sarris et al. conducted in vivo investigations using chemically induced tumors in Smyd3-deficient, Smyd3-wild type, and Smyd3-overexpressed mice (these results are described in paragraph one of the colorectal cancer section) [36]. After demonstrating the tumorigenic role of Smyd3 in vivo, Sarris et al. pursued to understand the mechanisms that prevented tumor formation in the Smyd3-deficient mice. DEN-induced tumors in wild-type mice showed significantly elevated mRNA expression levels of cell cycle regulators (*CcnA2, CcnD1, CcnE1* genes), and ChIP analysis confirmed that Smyd3 is recruited to the promoters of these cell cycle regulators (*CcnA2, CcnE1, CcnD1, Pcna,* and *Igfbp1*) [36]. However, mRNA levels of these target genes were not significantly affected in DEN-induced tumors of Smyd3-deficient mice. Based on this finding, the authors noted that Smyd3 may be necessary for the transcriptional activation of genes required for cellular proliferation in DEN-induced tumors [36]. Another critical mechanism that Sarris et al. explored was epithelial-mesenchymal transition (EMT), a key process in cancer initiation and metastasis [36]. There was a significant reduction of E-cadherin staining intensity in the liver/colon tumors of wild-type mice as compared to Smyd3-deficient mice. Furthermore, mRNA levels of transcription regulators (*Snai1, Snai2, Twist, Zeb1,* and *SOX4*) and EMT marker genes (*Fn1, Vimentin, Timp1, Mmp2, Mmp7, Mmp9, Mmp14*) were significantly increased in liver/colon tumors of wild-type mice when compared to Smyd3-deficient mice [36]. Lastly, the authors provided evidence that Smyd3 selects its transcriptional targets through multiple mechanisms: interaction with DNA directly, interaction with the RNA polymerase II machinery, and direct interaction with H3K4me3-modified nucleosomes. This study comprehensively investigated the genome-wide distribution of Smyd3 and provided insight into the role of Smyd3 in the development and progression of HCC [36].

Moreover, Wang et al. demonstrated that high SMYD3 expression predicted poor prognosis for patients with HCC and was found to be an independent prognostic factor for 5-year HCC tumor relapse [2]. Consistently with the two studies above, Wang et al. provided further evidence corroborating the role of SMYD3 in HCC cellular proliferation and invasion by demonstrating that SMYD3 enhanced the transcription of cyclin-dependent kinase 2 (CDK2) and matrix metalloproteinase-2 (MMP2) through tri-methylation of H3K4 at the corresponding promoter sites [2]. The findings point toward the role of SMYD3 in cellular proliferation (CDK2) and EMT (MMP2).

In another study, Zhou et al. demonstrated that HCC patients with SMYD3-positive expression had shorter overall and recurrence-free survival compared to those with negative SMYD3 expression [39]. Mass spectrometry analysis that was validated by co-immunoprecipitation (Co-IP) showed that SMYD3 overexpression led to the interaction of Ankyrin Repeat and KH Domain Containing 1 (ANKHD1) and H3K4me3, while SMYD3 knockdown inhibited this interaction [39]. Further, they confirmed the nuclear co-localization of H3K4me3 and ANKHD1 when SMYD3 was overexpressed, and both cytoplasmic and nuclear co-localization of SMYD3 and ANKHD1 [39]. These results highlight that ANKHD1 could be regulating the expression of SMYD3-controlled genes. Consistently, ANKHD1 positivity was significantly associated with large tumor size, microvascular invasion, multiple nodules, poor tumor differentiation, high TNM stage, and most importantly, shorter overall survival and recurrence-free survival [39]. This study uncovers the SMYD3-ANKHD1 interaction and underscores that ANKHD1 may regulate SMYD3-targeted gene expression in HCC.

The Hepatitis B Virus (HBV) is ubiquitously known as an inciting factor of HCC, and SMYD3 expression was found to be regulated by a Hepatitis B Virus X Protein, HBX, in HCC cells [40]. More specifically, Yang et al. showed that HBX upregulates the expression of SMYD3 mRNA and protein in HepG2 cells [40]. Interestingly, the authors found that C-MYC levels were increased in HBX-positive cells and that SMYD3 knockdown led to downregulation of C-MYC mRNA levels [40]. This study supports that HBX may regulate SMYD3, and that C-MYC may be a downstream target gene of SMYD3 [40].

Hayashi et al. further investigated the relationship between HBX and SMYD3 [41]. Interestingly, while Yang et al. showed that HBX transcriptionally upregulates SMYD3 [40], this study showed that HBX is also an interacting protein of SMYD3 [41]. As it had been previously reported that SMYD3 methylates MAP3K2 [27], the authors hypothesized that the SMYD3-HBX interaction may activate pathways downstream of MAP3K2 signaling, such as AP-1 signaling [41, 42]. Using a luciferase reporter assay, they demonstrated that co-expression of HBX and SMDY3 enhanced AP-1 signaling compared to SMYD3 expression alone [41]. These results support that the HBX-SMYD3 interaction may induce the activation of AP-1 signaling in HBV-infected HCC cells [41].

Chen et al. further elucidated the downstream effects of the HBX-SMYD3 interaction in HCC development and identified a novel long non-coding RNA (lncRNA), lncIHS, that was upregulated by HBX-induced overexpression of SMYD3 [43]. They showed that lncIHS can activate the AKT- and ERK-signaling pathways, as well as EMT features, such as invasion and migration of HCC cells [43].

In summary, these studies support that SMYD3 is an independent prognostic factor of survival in HCC. Mechanistically, it regulates the transcription of genes involved in cell proliferation, cell cycle, invasion and EMT pathways through tri-methylation of H3K4. Particularly in HBV-induced HCC, SMYD3 may directly interact with HBX to activate oncogenic pathways, such as the AKT-, ERK-, and AP-1 signaling, promoting the development of HCC in HBV-infected liver cells. The above support further investigation of SMYD3 as a therapeutic target in HCC.

#### Breast cancer

Through a bioinformatics interrogation of mRNA expression levels of all SMYD family members in breast cancer samples, Song et al. identified that SMYD3 was significantly upregulated in breast cancer patients in 27 different databases [44]. Specifically, SMYD3 was upregulated in medullary, invasive ductal, and invasive lobular carcinomas [44]. Additionally, SMYD3 expression was correlated with both estrogen and progesterone receptor positivity [44]. Furthermore, Kaplan–Meier curves showed that decreased SMYD3 mRNA expression was associated with improved relapse-free survival [44].

Hamamoto et al. also showed that SMYD3 mRNA expression is significantly upregulated in breast cancer tissues compared to normal counterparts [45]. Furthermore, SMYD3 knockdown resulted in decreased cellular proliferation of breast cancer cells [45]. Mechanistically, the authors showed that SMYD3 directly binds and upregulates *WNT10B*, a component of the Wnt/Beta-catenin pathway which is a commonly mutated pathway in many cancer types [45, 46].

Fenizia et. al demonstrated that SMYD3 functions in promoting EMT through the regulation of TGF-beta controlled EMT-specific transcription factors and mesenchymal gene expression [47]. They also demonstrated a TGF-beta-independent interaction between SMYD3 and SMAD3 [47], and that SMYD3 is required for TGF-beta induced SMAD3 recruitment at chromatin regulatory regions of EMT and mesenchymal genes [47]. It was also noted that SMYD3 transcripts appear to be elevated in all breast cancer subtypes, further demonstrating the importance of SMYD3 in breast cancer.

HER2 (EGFR2,ERBB2) amplified in approximately 18–25% of human breast cancers requires homodimerization or heterodimerization followed by autophosphorylation in order to be activated [48]. Yoshioka et al. demonstrated that SMYD3 tri-methylates HER2 at lysine 175 and that this methylation enhances HER2 homodimerization and subsequent activation [30]. Furthermore, SMYD3 knockdown reduced HER2 phosphorylation, while SMYD3 overexpression increased HER2 phosphorylation and activation [30]. These data highlight that in addition to its histone-mediated effects, the non-histone target functions of SMYD3 are critical to understanding its role in carcinogenesis.

Kunizaki et al. studied the interaction between SMYD3 and VEGFR1 in vitro in breast cancer cell lines (and colorectal and hepatocellular carcinoma cell lines) [26]. VEGFR, a receptor tyrosine kinase, functions primarily in angiogenesis and has been reported to function in tumor growth and progression [26]. The authors found that SMYD3 accumulates in the cytoplasm when cells are arrested at G0/G1, and cytoplasmic SMYD3 interacts with VEGFR1 [26]. The key finding of this study is that SMYD3 methylates VEGFR1 at lysine 831, and this methylation enhances the kinase activity of VEGFR1 via either a kinase domain conformation change or inhibition of a domain that suppresses VEGFR1 function [26].

Kim et al. investigated and demonstrated the role of SMYD3 in ER-mediated transcription [49]. When estrogen binds to ER, ER then binds to specific DNA sequences that are known as estrogen response elements (ERE), leading to transcriptional upregulation of specific genes [49, 50]. Kim et al. showed that SMYD3 interacts with ER and that SMYD3 functions as a transcriptional coactivator of ER, enhancing ER-mediated transcription [49]. Furthermore, SMYD3-dependent activation of ER correlates with H3K4 methylation at ER target genes and SMYD3 knockdown significantly attenuates expression of ER target genes [49].

Ren et al. studied the proliferative and cell cycle-specific effects of SMYD3 in breast cancer cells [51]. SMYD3 overexpression demonstrated induced enhanced cell proliferation and colony formation [51]. Conversely, SMYD3 knockdown inhibited cell proliferation. Further investigation highlighted that proliferation arrest was likely secondary to G1 growth phase arrest [51]. In SMYD3-depleted cells, PCR demonstrated decreased expression of Cyclin D1, CDK2, and CDK4, three key cell cycle regulators. Furthermore, the authors demonstrated that SMYD3 knockdown had a pro-apoptotic effect via increased expression of p53/p21 and downregulation of Bcl-2/Bax [51].

In summary, SMYD3 is overexpressed and associated with poor prognostic outcomes in breast cancer. Mechanistically, SMYD3 has been shown to interact and activate the HER2 receptor, estrogen receptor, VEGFR1, and SMAD3 in breast cancer cells, and it promotes cell proliferation and colony formation. These studies suggest that SMYD3 is a promising therapeutic target in breast cancer; however, further investigation is required to delineate its specific roles in triple-negative versus hormone-receptor positive breast cancer subtypes.

#### Gastric carcinoma

Liu et al. investigated the clinicopathologic role of SMYD3 expression in gastric carcinoma (GC) [52]. They discovered higher SMYD3 mRNA and protein expression levels in both GC tissues and cell lines compared to normal counterparts [52]. Clinically, positive SMYD3 expression was significantly associated with larger tumor size, increased lymph node metastasis, and advanced TNM stage [52]. Furthermore, patients with positive SMYD3 expression had a lower 5-year survival rate than those with negative expression [52].

Wang et al. demonstrated an epigenetic switch of cancer stem cells (CSCs) through SMYD3-dependent activation of the stem cell transcription factor Achaete-scute homolog 2 (ASCL2) in gastric carcinoma [53]. ASCL2 is regulated by the Wnt/Beta-catenin pathway, a pathway commonly implicated in carcinogenesis. ASCL2 is localized in the stem cells of the stomach and intestine, and it functions as a master regulator of stem cell maintenance. It is upregulated in gastrointestinal cancers and is associated with disease progression [53]. In this study, the authors demonstrated that ASCL2 + CSCs are Wnt-responsive and that depletion of ASCL2 in ASCL2 + cells leads to impaired self-renewal and tumorigenic capacity, suggesting that ASCL2 is an important regulator of CSCs in GC. They further demonstrate that SMYD3 mediates epigenetic activation of ASCL2 through tri-methylation of H3K4 at the gene locus, promoting self-renewal of ASCL2 + CSCs [53]. Data from these studies suggest a SMYD3-ASCL2 axis whereby Beta-catenin/TCF4-mediated transcriptional upregulation of SMYD3 leads H3K4me3-mediated ASCL2 activation and ultimately increases self-renewal and tumorigenicity in GC CSCs [53].

In another study, Liu et al. investigated the association between SMYD3 and signal transducer and activator of transcription 3 (STAT3) activation in 50 patients with gastric cancer [54–56]. STAT3 inhibition has been shown to attenuate cell viability and promote apoptosis in a variety of cancer models [57]. GC and GC cell lines have shown STAT3 upregulation, and this is thought to lead to increased cell proliferation by decreasing apoptosis via regulating Bcl-2, Bcl-xL, survivin, and cyclin D1 expression [54]. In this study, SMYD3, STAT3, and pSTAT3 expression levels were significantly higher in GC tissues than non-tumor tissues and positively correlated with each other [54]. The authors hypothesized that SMYD3 may contribute to GC carcinogenesis via STAT3 activation; however, relevant mechanistic studies are required to corroborate this hypothesis [54].

Wang et al. demonstrated that SMYD3 knockdown results in cell cycle arrest, specifically G2/M-phase arrest, in GC cells [58]. They showed that cell cycle arrest may be secondary to activation of the ATM-CHK2/p53-Cdc25C pathway, a key regulator of cell cycle control and DNA damage repair [58]. Upon activation, downstream mediators such as p53 and CHK2 are phosphorylated, activated, and interacted with downstream targets. The authors show that upon SMYD3 depletion, expression levels of ATM (total ATM and phosphorylated ATM), p53, CHK2, and p21 all increased, while CDK1 and cyclin B, both key effectors of the G2 phase, were downregulated [58]. These results highlight that SMYD3 could activate the ATM-CHK2/p53 pathway and lead to G2/M progression [58]. Lastly, the authors demonstrated that SMYD3 knockdown decreased cell migration and invasion, while SMYD3 overexpression had the opposite effect.

In summary, SMYD3 is overexpressed in GC and has been correlated with poor survival in GC patients. SMYD3 has been reported to function in key pathways for self-renewal and tumorigenicity of GC stem cells through activation of the cancer stem cell transcription factor ASCL2. Similarly to other cancer types, the above studies support that SMYD3 also regulates cell cycle progression, invasion, and metastasis in GC. Based on the above, SMYD3 merits further investigation as a potential therapeutic target in this disease.

#### Cervical cancer

Wang et al. investigated the role of SMYD3 in cervical carcinoma cell lines [59]. SMYD3 depletion decreased cell proliferation, colony formation, migration, and invasion, and increased apoptosis [59]. These results, like the functions of SMYD3 in other cancer subtypes, highlight the effects of SMYD3 on cell proliferation, migration, and invasion. More in-depth investigation of the role of SMYD3 in cervical carcinogenesis is required.

#### Prostate cancer

Lobo et al. investigated the role of SMYD3, as well as Ki67 (marker of cell proliferation) and EZH2, as independent predictive biomarkers in 189 consecutive diagnostic prostate biopsies [60]. The authors found that SMYD3, Ki67, and EZH2 independently predicted prostate cancer patient outcome adjusted for standard clinicopathologic parameters in their cohort [60].

Vieira et al. identified that SMYD3 knockdown attenuates cell proliferation and induces apoptosis in prostate carcinoma cells [61]. The attenuated cellular proliferation was correlated with downregulated c-MET, MMP-9, and NKX2.8, implicated in cell proliferation and migration [61]. In vivo mouse models demonstrated that SMYD3 knockdown reduced tumor growth [61]. Furthermore, the authors analyzed global methylation levels of various histone marks and found H4K20me3 predominantly affected by SMYD3 depletion in prostate cancer cell lines. Moreover, the authors provided evidence that SMYD3′s oncogenic properties in prostate cancer likely rely on its methyltransferase activity by showing that cell proliferation, apoptosis, and cell viability were not altered when sh-SMYD3 stably expressing cancer cells were transfected with enzymatically inactive SMYD3 [61]. Additionally, the authors identified that SMYD3-depleted prostate cancer cells show S phase arrest, and this correlates with increased expression of cyclin D2 (CCND2)*,* a key cell cycle regulator at the G1/S checkpoint that has been shown to be frequently silenced in prostate carcinomas [61, 62]. Primary prostate carcinoma expression profiles also demonstrated SMYD3 overexpression and CCND2 downregulation [61]. To investigate this further, a ChIP assay was performed to look at histone marks in the promoter region of CCND2. While no changes in H3K4 and H3K27 methylation marks were seen, there was a significant decrease in the H4K20me3 repressive mark in sh-SMYD3 prostate cancer cells, providing further evidence that SMYD3 expression may repress CCND2 expression via H4K20 tri-methylation in prostate cancer [61].

Liu et al. investigated the epigenetic regulation of the androgen receptor (AR) in prostate cancer [63]. AR is the key signaling pathway in both normal prostate and prostate cancer growth [63, 64]. SMYD3 depletion resulted in a significant decrease in cell proliferation by S phase arrest, and colony formation was also significantly reduced [63]. In vivo experiments demonstrated decreased tumor growth, decreased cell proliferation, and increased apoptosis in SMYD3-depleted mice [63]. They then focused on mechanisms whereby SMYD3 carries out its oncogenic effects in prostate cancer. They showed that SMYD3 knockdown results in AR downregulation on the mRNA and protein levels [63]. To further interrogate the SMYD3-AR interaction, the authors conducted ChIP assays showing that SMYD3 binds to the AR promoter and SMYD3 knockdown leads to decreased H3K4me2 and H3K4me3 deposition on the AR promoter site [63].

In summary, SMYD3 is commonly overexpressed in prostate cancer and is an independent predictor of poor outcome. SMYD3 promotes cellular proliferation, and mechanisms include H4K20-mediated repression of cyclin D2, as well as H3K4-mediated upregulation of the AR. These preliminary results provide evidence that SMYD3 may be a rational target for prostate cancer.

#### Pancreatic cancer/lung cancer

Zhu et al. focused on clinicopathologic associations of SMYD3 in pancreatic cancer [65]. They identified that SMYD3 expression positively correlated with tumor size, TNM stage, perineural invasion, and lymph node metastasis in pancreatic adenocarcinoma [65]. Further, patients with positive expression of SMYD3 had significantly shorter survival than those with negative expression, and SMYD3 was identified as an independent predictive factor for overall survival [65].

Pancreatic adenocarcinoma (PDAC) is almost ubiquitously initiated by a mutation in the Ras pathway [27]. Mazur et al. aimed to explore the role of PKMTs in Ras-driven cancers and chose SMYD3 as it had the highest level of expression in their analysis and has been implicated in the Ras pathway [27]. PDAC is thought to arise from the differentiation of acinar cells into duct-like cells upon activation of Ras signaling [66, 67]. The authors showed via a Smyd3-mutant mouse model that Smyd3 was required for acinar cell differentiation to occur [27, 67]. Further, they identified that Smyd3 depletion reduced the appearance of pancreatic intra-epithelial neoplasia (PanIN) seen with *Kras* activation in vivo, reduced phosphorylated ERK1/2 levels (pERK1/2, downstream effector of Ras and PDAC biomarker) and MUC5 (a marker of PanIN), and extended the mouse life span [27]. From this, the authors determined that SMYD3 is necessary for pancreatic cancer initiation via the K-Ras pathway [27].

Further, Mazur et al. also studied these findings in lung adenocarcinoma, a malignancy frequently driven by activation of the Ras pathway with high levels of SMYD3 expression [27]. In a lung adenocarcinoma Kras mutant mouse model, they demonstrated that Smyd3-deficient mice had significantly smaller and less advanced tumors that controls [27]. Additionally, histologic evidence demonstrated that loss of Smyd3 prevented transition from adenoma to adenocarcinoma in these lung specimens [27]. Further, Smyd3 depletion resulted in lower detection of pERK1/2, a key finding as amplification of Ras/MEK/ERK signaling correlates with lung carcinogenesis [27].

In vitro, Mazur et al. identified SMYD3 knockdown in three cell lines (2 lung adenocarcinoma, 1 PDAC) resulted in decreased cellular proliferation and inhibited anchorage-independent growth [27]. As almost all SMYD3 in these samples was cytoplasmic, the authors performed a large biochemical screen for cytoplasmic substrates of SMYD3 and found MAP3K2, a component of the MAP kinase pathway [27]. They identified that MAP3K2 can be mono-, di-, or tri-methylated by SMYD3 at lysine 260 and this is lost when SMYD3 is knocked down [27]. However, as the intrinsic kinase activity of MAP3K2 is not altered by methylation, the authors hypothesized that the alteration in MAP3K2 function in the absence of SMYD3 is secondary to a protein–protein interaction [27]. A proteomics assay demonstrated that six candidate proteins bind to unmethylated MAP3K2 but are blocked from binding to tri-methylated MAP3K2. Three of the 6 proteins are members of the protein phosphatase 2A (PP2A) complex, which is well known to inactivate members of the MAP kinase signaling pathway [27, 68, 69]. This finding demonstrates that SMYD3-dependent MAP3K2 tri-methylation effectively blocks MAP3K2 from inactivation by PP2A [27].

The role of SMYD3 in non-small cell lung cancer (NSCLC) has been further investigated by Li et al. [70]. They first performed IHC on 155 pairs of NSCLC and adjacent normal tissue samples and showed that SMYD3 was significantly upregulated in NSCLC samples when compared to normal tissue samples [70]. Li et al. found that high SMYD3 expression was significantly associated with a never-smoked history, advanced pathological stage, larger tumor size, the presence of lymphovascular invasion, pleural invasion, and distant metastasis [70]. Furthermore, disease-free survival and overall survival were poorer in patients with high SMYD3 expression compared to low SMYD3 expression [70]. Next, Li et al. showed that SMYD3 knockdown decreased cell proliferation, while SMYD3 overexpression increased cell proliferation in NSCLC cell lines [70]. SMYD3 depletion was shown to increase apoptosis, specifically through increased expression of Bim, Bak, and Bax, and a decreased expression of BCL-2 and Bcl-xl [70]. Also, SMYD3 depletion sensitized NSCLC cells to cisplatin-induced apoptosis, while SMYD3-overexpressed cells were more resistant to cisplatin, highlighting the role of SMYD3 in cisplatin resistance in NSCLC [70]. Furthermore, SMYD3 knockdown significantly decreased MMP-2 and MMP-9 expression, migration, and invasion in NSCLC cells [70].

Additionally, a recent study conducted by Zhang et al. showed that SMYD3 expression levels were higher in NSCLC KRAS-mutated patient-derived xenografts that responded to combination treatment with a MEK + BCL-X/BCL-2 inhibitor (inhibitor that induces apoptosis) compared to treatment-resistant tumors [71]. The authors hypothesize that SMYD3 expression was increased in the sensitive tumors due to SMYD3-dependent methylation of MAP3K2 [27]. MAP3K2 is a member of the RAS-driven tumorigenesis pathway, so when SMYD3 is overexpressed, there might be greater activation of the RAS pathway via MAP3K2 and thus, greater sensitization of these tumors to MEK inhibition [71]. However, mechanistic studies to provide evidence for this hypothesis were not performed [71].

In summary, SMYD3 is critical for the activation of MAP3K2, a key kinase in the Ras-activated MAP signaling pathway, in both lung and pancreatic cancers. Furthermore, SMYD3 is associated with advanced stage and poor survival in NSCLC, and promotes cell proliferation, invasion, and chemotherapy resistance phenotypes. These data support that SMYD3 could serve as an important therapeutic target in NSCLC.

#### Ovarian cancer

Zhang et al. identified that SMYD3 copy number was higher in ovarian serous cystadenocarcinoma than normal ovarian tissue (TCGA) [72]. IHC demonstrated that SMYD3 tended to be cytoplasmic in ovarian tumor samples, and that SMYD3 expression gradually increased from normal ovaries, fallopian tubes, primary cancer lesions, to metastatic lesions [72]. High SMYD3 expression was significantly correlated with metastasis, advanced stage and ascites [72]. Furthermore, high SMYD3 expression correlated with significantly lower progression-free survival and overall survival compared with patients with low SMYD3 expression [72].

Zhang et al. demonstrated that SMYD3 upgrades the migratory capacity of ovarian cancer cells and promotes ovarian cancer metastasis using in vivo mouse models [72]. Mechanistically, they showed that SMYD3 directly interacts with p53 via its post-SET domain and induces its destabilization by promoting its ubiquitination and proteasomal degradation. More specifically, SMYD3 expression downregulated p53 protein levels and promoted translocation of p53 from the nucleus to cytoplasm [72]. Next, the authors identified these effects were not due to SMYD3-mediated methylation of p53 [72]. As ubiquitination of p53 has been shown to change p53′s cellular location, the authors hypothesized that SMYD3 may induce ubiquitination of p53. Indeed, they found that SMYD3 promotes ubiquitination independent of MDM2, an E3-ubiquitin ligase [72]. Accordingly, mass spectrometry analysis demonstrated an interaction between SMYD3 and UBE2R2, a ubiquitin-conjugating enzyme [72]. Based on this finding, the authors posited that SMYD3 may function as a E3 ubiquitin ligase [72] and showed that the concurrent overexpression of SMYD3 and UBE2R2 significantly promoted ubiquitination and degradation of p53 [72]. This offers a novel mechanism whereby SMYD3 promotes the metastatic potential of ovarian cancer cells through ubiquitination and destabilization of p53, a critical regulator of carcinogenesis, independent of its methyltransferase activity.

Lyu et al. further investigated the role of SMYD3 in ovarian cancer metastasis [75]. Epithelial ovarian cancer frequently metastasizes to the peritoneal cavity, and multicellular ‘spheroids’ are commonly observed in the malignant ascitic fluid. The authors showed that SMYD3 is overexpressed in ovarian cancer spheroids, and that SMYD3 is critical to their invasive capacity. SMYD3 knockdown suppressed the adhesion and invasion of ovarian cancer spheroids. There also was concurrent downregulation of ITGB6 and ITGAM integrins, two genes important for cellular adhesion. Inhibition of ITGB6 and ITGAM directly also resulted in decreased ovarian cancer spheroid adhesion and invasion, and this was restored with re-expression of SMYD3, ITGB6, and ITGAM. ChIP assays showed increased SMYD3 and H3K4me3 promoter binding at the *ITGB6* and *ITGAM* gene loci in ovarian cancer spheroids and decreased binding with SMYD3 knockdown.

In another study, Jiang et al. showed that SMYD3 can increase cellular proliferation by accelerating the S phase [73]. Conversely, SMYD3 knockdown resulted in decreased ovarian cancer cell proliferation [73]. SMYD3 knockdown resulted in decreased mRNA levels of CCNA2, CCNB2, CCND2, CDK1, and CDK2, and increased mRNA levels of WEE1 [74], leading to S phase arrestin SMYD3-depleted ovarian cancer cells [73]. Additionally, SMYD3 depletion induced apoptosis and downregulation of Baculoviral IAP Repeat-Containing 3 (BIRC3), an inhibitor of apoptosis, as well as increased CD40L (pro-apoptotic) expression [73]. ChIP assays showed that SMYD3 promoted BIRC3 expression through H3K4me3 and inhibited CDKN2A expression through H4K20me3 [73]. Overall, these results highlight that SMYD3 promotes ovarian carcinogenesis through the transcriptional regulation of multiple cell cycle regulators and by inhibiting apoptosis through the regulation of antiapoptotic factors, such as BIRC3.

In summary, the functions of SMYD3 in ovarian carcinogenesis have been studied both clinicopathologically and mechanistically. SMYD3 has been shown to directly interact and affect the stability of p53, alter cell cycle progression and apoptosis, and promote ovarian cancer metastasis by directly regulating the transcription of integrins. From these studies, SMYD3 may be a rational therapeutic target in the treatment of ovarian cancer.

#### Esophageal cancer

Zhu et al. investigated SMYD3 expression by IHC in a tissue microarray from 131 patients with esophageal squamous cell carcinoma (ESCC) [76]. Analysis showed that ESCC patients with high SMYD3 expression demonstrated significantly poorer overall survival [76]. Increased SMYD3 expression was associated with lymph node metastasis and was an independent prognostic factor of poor overall survival [76]. Furthermore, knockdown of SMYD3 suppressed ESCC cell proliferation, migration, and invasion in vitro and inhibited local tumor invasion in vivo*.* The authors further demonstrated that SMYD3 depletion decreased the expression of Ezrin (EZR, member of a cytoskeleton protein family) and LOXL2 (member of the lysyl oxidase family), two genes that are important for proliferation, migration, and invasion in ESCC [76]. Furthermore, ChIP assays demonstrated that SMYD3 binds to the promoter regions of both *EZR* and *LOXL2* [76]. Lastly, IHC staining of ESCC samples demonstrated a positive correlation between SMYD3 expression and EZR/LOXL2 protein levels [76].

Zhang et al. further studied the interaction of SMYD3 and EZR in ESCC via a long non-coding RNA (lncRNA), EZR-AS1 [77]. The authors identified a natural EZR antisense lncRNA, EZR-AS1 [77]. Their work shows that lncRNA EZR-AS1 regulates and activates the transcription and expression of EZR, and ultimately ESCC cellular migration by interacting with both RNA polymerase and SMYD3 [77]. EZR-AS1 recruits SMYD3 to SMYD3 binding sites in the GC-rich region downstream of the EZR promoter, leading to SMYD3-targeted H3K4 tri-methylation, while the SMYD3-EZR AS1 interaction that enhances EZR transcription and expression to increase migration of ESCC cells [77].

In another interesting study, Wang et al. investigated the effects of a SMYD3 variable number tandem repeat (VNTR) polymorphism in the promoter region of the SMYD3 gene on ESCC cancer progression risk [78]. They analyzed 567 patients and 567 control subjects in a Chinese cohort [78]. From their analysis, they found that subjects having the SMYD3 3/3 VNTR genotype had a threefold increased risk of ESCC when smoking, but that there was no increased risk of ESCC among non-smokers [78]. Interestingly, the tandem repeat sequence of ‘CCGCC’ in the SMYD3 promoter region is a binding site for E2F1, a transcription factor involved in key processes such as cell cycle, DNA synthesis, and DNA repair [79]. Furthermore, the major allele of the three repeats motif (SMYD3 3/3, high risk) confers increased binding affinity to E2F1 when compared to the two repeats motif (2/2, low risk) [78]. The authors speculated that the reason for the increased ESCC risk in smokers may involve a gene–environment interaction between the SMYD3 polymorphism and tobacco smoke.

Dong et al. investigated the interaction between SMYD3 and retinoblastoma protein-interacting zinc finger gene 1 (RIZ1) [80]. RIZ1 is a member of the nucleoprotein methyltransferase family and is known to methylate H3K9 [80]. It is thought to have a tumor suppressive effect in many cancer types, including ESCC [80]. In this study, the authors analyzed ESCC tissues and non-cancerous esophageal tissue and identified that SMYD3 expression was significantly increased in the ESCCs [80]. MTT assays demonstrated decreased cellular proliferation in SMYD3-depleted ESCC cells [80]. Furthermore, RIZ1 mRNA and protein expression levels were significantly increased in SMYD3-depleted ESCC cells, providing evidence toward a possible SMYD3-RIZ1 downstream pathway [80].

In summary, SMYD3 is overexpressed and is associated with poor survival in ESCC patients. A specific *SMYD3* promoter VNTR has also been associated with a higher risk for tobacco-induced ESCC. SMYD3 has been shown to directly bind on the gene loci of *EZR* and *LOXL2* which promote proliferation and invasion in ESCC. The above studies suggest that SMYD3 is important in ESCC and merits further investigation as a therapeutic target in this disease.

#### Bladder cancer

Wang et al. investigated the interaction of SMYD3 with the insulin-like growth factor-1 receptor (IGF-1R) and the AKT/mTOR pathway in bladder cancer [81]. First, SMYD3 expression was higher in bladder cancer samples compared to normal matched tissues, and it positively correlated with tumor stage and lymph node metastasis. TCGA database analysis also showed that higher SMYD3 mRNA correlated with a significantly shorter progression-free survival. SMYD3 depletion inhibited cell proliferation and colony formation, invasion, and migration in bladder cancer cell lines, as well as xenograft tumor formation in vivo. Mechanistically, the authors found that SMYD3 directly bound to the promoter of *IGF-1R*, an activator of the AKT/mTOR pathway, and activated its transcription through tri-methylation of H3K4. SMYD3 depletion also resulted in diminished AKT/mTOR signaling, which was hypothesized to be secondary to decreased IGF-1R transcriptional activation. Interestingly, the authors also found that E2F-1, a downstream mediator of the AKT pathway, directly binds and transcriptionally activates the *SMYD3* promoter. These experiments support that SMYD3 participates in a positive feedback loop between the IGF-1R, AKT/mTOR and E2F-1 pathways,

Wu et al. further investigated the role of SMYD3 in bladder cancer [83]. The authors first demonstrated that SMYD3 expression was higher in bladder cancer cell lines than a normal bladder cell line. They subsequently showed that SMYD3-depleted bladder cancer cells have decreased H3K4me2 and H3K4me3 expression levels without a significant change in H3K4me1 expression relative to controls. Similar to Wang et. al.’s experiments, Wu et al. found that SMYD3 depletion inhibited cell proliferation, colony formation, and cell migration and invasion, and increased apoptosis. These findings suggest that SMYD3 may play a critical role in bladder cancer oncogenesis; however, more mechanistic exploration is needed.

Shen et al. investigated the role of SMYD3 in the regulation of autophagy activation in bladder cancer. First, they found that SMYD3 expression correlated with lymph node metastasis and shorter overall survival [84]. The authors also showed that ectopic expression of SMYD3 promotes cell proliferation and invasion in a human bladder cancer cell line [84]. Using publicly available expression datasets, Shen et al. identified that the BCL2-associated transcription factor 1 (BCLAF1), a protein important in autophagy pathways [85, 86], might be regulated by SMYD3 [84]. SMYD3 expression correlated with BCLAF1, and SMYD3 bound to the *BCLAF1* promoter region and regulated *BCLAF1* via H3K4 methylation [84]. Lastly, BCLAF1 was shown to activate autophagy in bladder cancer cells, underscoring that SMYD3 plays a critical role in bladder cancer oncogenesis and autophagy activation via H3K4me2/me3-mediated regulation of *BCLAF1* [84].

In summary, SMYD3 plays critical roles in bladder cancer oncogenesis, such as interactions with IGF-1R and AKT/mTOR in a possible feedback pathway, cell cycle and apoptotic regulation, and autophagy activation via BCLAF1 regulation. SMYD3 may represent a possible therapeutic target in the treatment of bladder cancer.

#### Glioma

Dai et al. investigated the oncogenic role of SMYD3 in malignant glioma [87]. First, IHC showed that SMYD3 is overexpressed human glioma samples but not in normal brain tissues. Furthermore, higher SMYD3 expression levels correlated with higher tumor grade and worse survival. Additionally, SMYD3 expression was higher in high pathologic grade gliomas when compared to low pathologic grade [87]. Kaplan–Meier analysis showed significantly longer survival in patients with gliomas with low SMYD3 expression as compared to patients with high SMYD3 expression [87]. SMYD3 depletion resulted in decreased cell proliferation and colony formation in human glioma cell lines. Furthermore, SMYD3 expression was shown to promote tumorigenesis in vivo [87]. As p53 had previously been shown to play an important role in malignant gliomas [88], the authors examined whether SMYD3 affected p53 in human glioma cell lines and showed that SMYD3 depletion increased p53 protein levels, and conversely, SMYD3 overexpression decreased p53 and protein levels [87]. The antiproliferative phenotype induced by SMYD3 depletion was mediated by p53 overexpression, as depletion of p53 in shSMYD3 stably expressing cell lines reversed this antiproliferative effect [87]. Additionally, a negative correlation between SMYD3 and p53 expression was observed in the human glioma samples. In summary, SMYD3 may play a role in the tumorigenesis of malignant glioma, but further mechanistic investigation is merited.

### A review of currently available SMYD3 inhibitors

The role of SMYD3 role in malignant proliferation, migration, invasion, and progression has been extensively studied in many cancer types, as demonstrated above. In normal tissues, SMYD3 expression is low or absent, while in cancers, SMYD3 is significantly overexpressed [89]. This is ideal from a drug discovery standpoint, as it means that SMYD3 inhibition could be an effective anti-cancer target with few off-target effects [89]. In this section, we will review current efforts to generate SMYD3 inhibitors for future pharmacologic therapy.

**Fig. 2:**
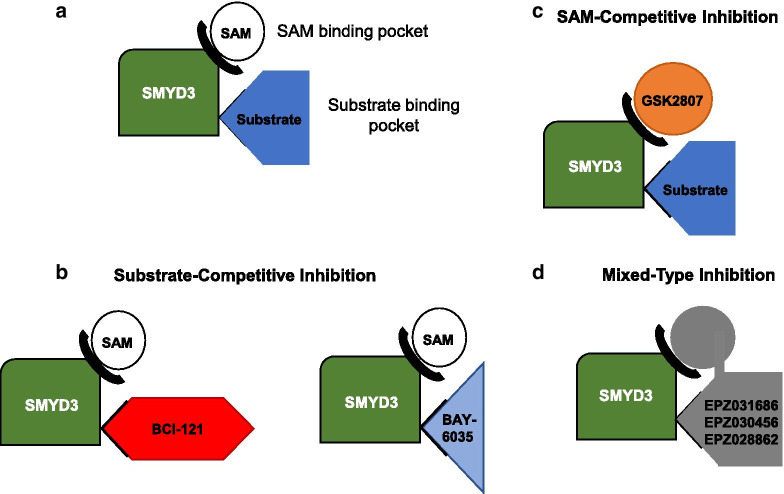
Main mechanisms of drug-mediated SMYD3 inhibition. **a** SMYD3 protein graphic illustrating two critical binding areas for inhibitors, the SAM- (S-adenosyl-methionine), and substrate-binding pockets. **b** Substrate-competitive inhibition. BCI-121 and BAY-6035 bind to the substrate-binding pocket of SMYD3. **c** SAM-competitive inhibition. GSK2807 binds to the SAM-binding pocket and inhibits binding of SAM to SMYD3. **d** Mixed-type inhibition. EPZ031686, EPZ030456, and EPZ028862 inhibit both the substrate- and SAM-binding pockets of SMYD3

While other PKMT inhibitors have begun to enter clinical trials (DOT1L, EZH2, and PRMT5), SMYD inhibitors are still in the preclinical phase [89]. There are currently 7 SMYD3 small-molecular inhibitors available as of April 2019: BCI-121, EPZ030456, EPZ031686, GSK2807, EPZ028862, BAY-6035, and tetrahydroacridine compounds [89]. We will discuss each inhibitor individually below. The specific medicinal chemistry of each inhibitor is outside the scope of this review. Figure 2 illustrates the basic mechanisms of action of these SMYD3 inhibitors.

### BCI-121

Its mechanism of action is via substrate competitive inhibition of SMYD3 [89]. At 100 uM, BCI-121 was shown to impair target methylation (H3K4 and H4K5) while leaving non-targets (H3K27) unaffected [89]. Additionally, BCI-121 was shown to have an anti-proliferative cellular effect in vitro in two colon adenocarcinoma cell lines [89]. At 100 uM, BCI-121 was also able to reduce the expression of SMYD3-target genes in the colon adenocarcinoma cell lines, indicating the drug’s ability to prevent SMYD3 recruitment at promoter sites [89]. BCI-121 has not been tested in vivo.

### GSK2807 (GlaxoSmithKline)

Its mechanism of action is SAM competitive inhibition and non-competitive inhibition of MAP3K2, a non-histone target of SMYD3 [89]. Inhibition of SMYD3 by GSK2807 was found to be selective for SMYD3 when screened against other PKMTs [89]. Furthermore, GSK2807 was found to be 24 times less active toward SMYD2, a closely related SMYD family enzyme [89]. GSK2807 has not been tested in vivo.

### EPZ031686 & EPZ030456 (Epizyme)

EPZ031686′s mechanism of action is non-competitive inhibition of SAM and MAP3K2, while EPZ030456′s mechanism of action is a mixed-type inhibition of SAM and MAP3K2, one of SMYD3′s non-histone substrates [89]. Both compounds were the first two SMYD3 inhibitors with double-digit nanomolar activities in biochemical and cellular assays and with pharmacokinetics to be tested in vivo [89]. Both have been shown to be highly selective for SMYD3 when tested against other PKMTs in vitro [89]. After 50 mg/kg PO administration of EPZ031686 to mice, plasma concentration remained above IC_50_ for more than 12 h, highlighting its possible efficacy in future in vivo models. EPZ030456, however, could not be tested in vivo due to its lower solubility [89].

### EPZ028862 (Epizyme)

Its mechanism of action is mixed-type inhibition toward SAM and non-competitive inhibition of MAP3K2 [89]. In vitro, it showed equal activity in biochemical and cellular assays [89]. In vivo, EPZ028862 showed favorable pharmacokinetics. EPZ028862 showed selectivity for SMYD3 when tested against other PKMTs including SMYD2 [89]. No antiproliferative activity was seen in non-small cell lung cancer and other lung cancer cell lines with and without KRAS mutations [89]. Additionally, no effect on cell growth was observed in a panel of other cancer cell lines, neither with EPZ028862 alone or in combination with MAP2K1 (MEK1) inhibitor trametinib [89]. This conflicts with findings suggested by Mazur et al. described above [27].

### BAY-6035 (collaboration between SGC and Bayer AG)

Its mechanism of action is substrate-competitive inhibition [89]. While limited information was available at time of publication of the review, BAY-6035 is reported to be highly selective for SMYD3 inhibition in both in vitro and cell-based experiments [89].

### Tetrahydroacridine compounds

This is a novel class of inhibitors that inhibit SMYD3 irreversibly via covalent modifications, specifically a nucleophilic aromatic substitution reaction [90]. In vitro, some of these compounds had an antiproliferative effect on the HepG2 cell line, a hepatocellular carcinoma cell line [90]. Further investigation into the in vivo effects of these compounds is necessary, but early data are promising [90].

## Conclusions

In this review, we provide an overview of the literature pertaining to SMYD3, its reported histone and non-histone functions, implications in cancer, and drug discovery possibilities. SMYD3, via both histone-specific and non-histone-specific interactions, plays critical roles in cell cycle alteration, apoptosis, and EMT, which influence cell proliferation, migration, invasion, and metastasis. While many mechanisms of SMYD3 have been reported, its genome-wide distribution in human cells, as well as the full spectrum of its substrates, is still elusive. Furthermore, it will be important to gain a clearer understanding of its histone substrates; current knowledge supports that SMYD3 di- and tri-methylates H3K4, an activating mark; however, H4K20, a repressive mark, may be a predominant substrate depending on the cell context. Despite these deficiencies in our knowledge of its function, current research points to the promise of SMYD3 as a therapeutic target in the treatment of cancer.

## Data Availability

Not applicable.

## Abbreviations

SMYD3: SET and MYND-Domain Containing 3
H3K4: Histone 3 Lysine 4
PKMT: Protein Lysine Methyltransferase
H3K9: Histone 3 Lysine 9
H3K27: Histone 3 Lysine 27
H4K5: Histone 4 Lysine 5
H4K20: Histone 4 Lysine 20
SMYD: SET and MYND-Domain
SET: Su(var)3–9, Enhancer-of-zeste and N-terminal Trithorax domain
MYND: Myeloid-Nervy-DEAF1 domain
CTD: C-Terminal Domain
TPR: Tetratricopeptide Repeats (TPR)
ChIP: Chromatin immunoprecipitation
SAM: S-adenosyl-L-methionine
ER: Estrogen Receptor
HER2: Human epidermal growth factor receptor 2
HSP90: Heat Shock Protein 90
CRC: Colorectal Cancer
HCC: Hepatocellular Carcinoma
DEN: Diethylnitrosamine
DMH/DSS: 1,2-Dimethylhydrazine/dextran sodium sulfate
qMSP: Quantitative methylation-specific PCR
LN: Lymph node
TCGA: The cancer genome atlas
TNM: Tumor/nodes/metastasis staging
IHC: Immunohistochemistry
EMT: Epithelial-mesenchymal transition
AFP: Alpha fetoprotein
CDK2: Cyclin-depending kinase 2
MMP: Matrix metalloproteinase-2
Co-IP: Co-immunoprecipitation
ANKHD1: Ankyrin repeat and KH domain containing 1
AP-1: Activator protein 1
HBX: Hepatitis B Virus X Protein
lncRNA: Long non-coding RNA
GC: Gastric carcinoma
CSCs: Cancer stem cells
ASCL2: Achaete-scute homolog 2
STAT3: Signal transducers and activators of transcription 3
CCND2: Cyclin D2
AR: Androgen receptor
PDAC: Pancreatic adenocarcinoma
PanIN: Pancreatic intra-epithelial neoplasia
PP2A: Protein phosphatase 2A
NSCLC: Non-small cell lung cancer
EGFR: Epidermal growth factor receptor
BIRC3: Baculoviral IAP repeat containing 3 (BIRC3)
ESCC: Esophageal squamous cell carcinoma
EZR: Ezrin
VNTR: Variable number tandem repeat
RIZ1: Retinoblastoma protein-interacting zinc finger gene
IGF-1R: Insulin-like growth factor-1 receptor
BCLAF1: BCL2-associated transcription factor 1
